# Case Report: Lipoprotein Glomerulopathy Complicated by Atypical Hemolytic Uremic Syndrome

**DOI:** 10.3389/fmed.2021.679048

**Published:** 2021-06-02

**Authors:** Lara Kollbrunner, Patricia Hirt-Minkowski, Javier Sanz, Elena Bresin, Thomas J. Neuhaus, Helmut Hopfer, Andreas W. Jehle

**Affiliations:** ^1^Department of Internal Medicine, Hirslanden Klinik St. Anna, Lucerne, Switzerland; ^2^Transplantation Immunology and Nephrology, University Hospital Basel, Basel, Switzerland; ^3^Division of Human Genetics, University Hospital of Bern, Bern, Switzerland; ^4^Istituto di Ricerche Farmacologiche Mario Negri Istituto di Ricovero e Cura a Carattere Scientifico (IRCCS), Bergamo, Italy; ^5^Department of Pediatrics, Lucerne Children's Hospital, Cantonal Hospital Lucerne, Lucerne, Switzerland; ^6^Institute for Pathology, University Hospital Basel, Basel, Switzerland

**Keywords:** case report, lipoprotein glomerulopathy, thrombotic microangiopathy, atypical hemolytic uremic syndrome, nephrotic syndrome, apolipoprotein E, complement factor-H related 1

## Abstract

Lipoprotein glomerulopathy (LPG) is a rare inherited disease caused by mutations in the APOE gene, encoding apolipoprotein E (apoE). Atypical hemolytic uremic syndrome (aHUS) is a thrombotic microangiopathy (TMA) characterized by overactivation of the alternative complement pathway. Here we report the case of a 21-year-old man with LPG who developed aHUS. A functional complement assay demonstrated an overactivation of the complement system. Complementary genetic analysis revealed a homozygous aHUS risk allele for complement factor-H related 1 (CFHR1), CFHR1^*^B. To the best of our knowledge, this is the first report of an aHUS in a patient with LPG.

## Introduction

Lipoprotein glomerulopathy (LPG) is a rare inherited renal disease caused by mutations in the APOE gene, encoding apolipoprotein E (apoE) (1). Approximately 200 LPG cases have been reported worldwide, the majority in Asian populations (2). LPG is an autosomal dominant disorder with incomplete penetrance (1, 3). Gene analysis discovered various mutations of APOE. Most of them are missense variants close to the LDL receptor binding site. However, mutants far from the binding site and mutants involving deletion of several amino acids are also reported (4). The clinical manifestation and histological findings seem to be common with different APOE variants (1). A kidney biopsy is essential for LPG diagnosis (3). Pathognomonic histologic features include glomerular capillary lipid thrombi without significant infiltration by foamy macrophages (5, 6). Clinically the disease manifests with proteinuria or nephrotic syndrome with or without an abnormal lipid profile and may gradually progress to chronic renal failure (1).

Atypical hemolytic uremic syndrome (aHUS) is a thrombotic microangiopathy (TMA) characterized by overactivation of the alternative complement pathway (7). Complement factor H (CFH) is the primary inhibitor of this pathway (**Figure 2A**). The complement factor H-related (CFHR) gene family encodes a group of plasma proteins genetically and structurally related to CFH. The risk allotype CFHR1^*^B (**Figure 2B**), resulting from a gene conversion event between CFH and CFHR1, is strongly associated with aHUS. In this report, we present a case of LPG with aHUS.

## Case Description

**Figure 1:**
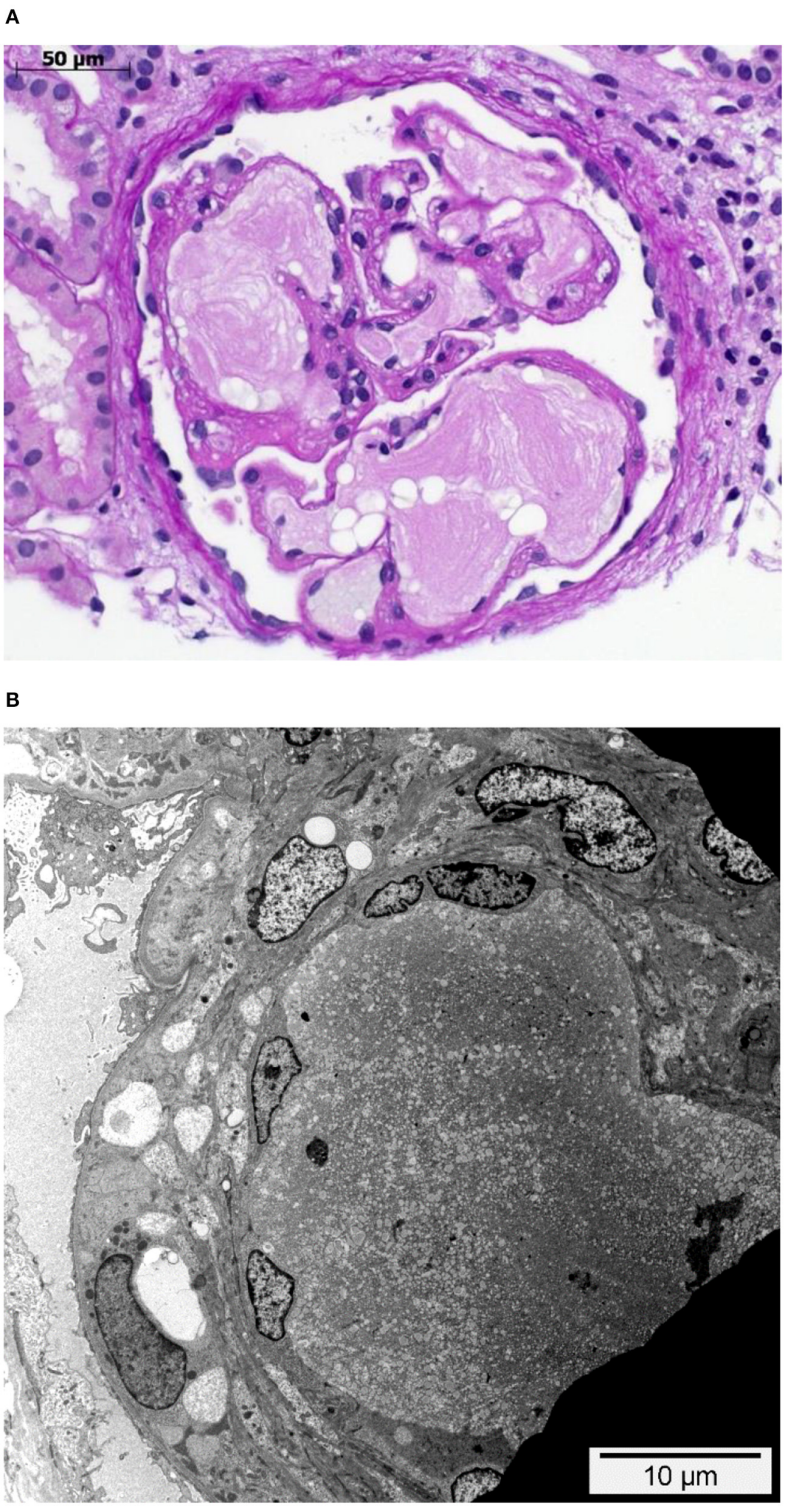
Glomerulus with intraglomerular lipoprotein thrombi. **(A)** Glomerulus with dilated glomerular capillaries containing characteristic acellular, lamellated, intracapillary lipoprotein thrombi. PAS staining. **(B)** Dilated peripheral glomerular capillary containing a lipoprotein thrombus with its characteristic electron microscopic appearance. While the endothelial cells on the left-hand side are visible and slightly enlarged, they are not detectable in the upper right side. Transmission electron microscopy.

A 21-year-old swiss-indonesian man was referred to our nephrology clinic in June 2019. He had end-stage renal disease with a creatine value of 907 μmol/l (Table 1). Previously, at the age of 14 years, an asymptomatic, increased blood pressure of 158/102 mmHg was discovered during a school-based examination, and he was investigated at the children's hospital of Lucerne, Switzerland. His medical history revealed swollen eyelids in the morning for the last 3 years. Clinical examination was regular except for bilateral ankle edema. Further workup identified a nephrotic syndrome with a protein to creatine ratio of 586 mg/mmol, a serum albumin concentration of 27 g/L, total cholesterol of 9.5 mmol/l (Table 1), and microhematuria with 30% dysmorphic erythrocytes. Screening for hepatitis B and C was negative, and autoantibodies (antinuclear antibodies, anti dsDNA antibodies, antineutrophil cytoplasmic antibodies, and antiglomerular basement membrane antibodies) were negative. Echocardiography showed mild left ventricular hypertrophy. The renal biopsy established the diagnosis of LPG. It showed glomerular capillary dilatations with lipoprotein thrombi with lamella formation (Figure 1A). No fibrin thrombi, fragmented erythrocytes or fibrinoid necrosis suggestive of aHUS was seen. Chronic changes in the form of secondary focal segmental scleroses and mild interstitial fibrosis with tubular atrophy were already present. By immunohistochemistry, mild to moderate unspecific depositions of IgM and C5b-9 within the glomeruli were detected. IgA, IgG, and C3c were negative within the glomeruli. Transmission electron microscopy showed the characteristic concentrically layered and vacuolated lipoprotein thrombi (Figure 1B). Analysis of APOE revealed E3/E3 genotype. Sequencing of the APOE gene detected a heterozygous 9-bp deletion in exon 4 (NM_000041.3: c.480_488del (p.Leu162_Lys164del, traditional nomencalture: 142_144del)), resulting in a 3-amino acid deletion in a region involved in receptor binding of the apo E molecule and previously associated with LPG (10, 11). In the literature, this mutation is often named APOE Tokyo/Maebashi (1). A grandfather of the patient in Indonesia received a kidney transplant at the age of about 60 years. Otherwise, the family history brought to light no renal disease in either of the patient's parents. The patient has no siblings. Therapy was started with an ACE-inhibitor (enalapril 10 mg/d) and a lipid-lowering agent (atorvastatin 20 mg/d). As the patient emigrated to Indonesia soon after the diagnosis, medical follow-up was lost.

**Table 1:** Laboratory values.

	**At diagnosis of LPG 2012**	**At admission** ** 2019**	**8-days after**	**12-days after**
**Blood**				
*Hematology*				
Hemoglobin (140–180 g/L)	127 g/L	84 g/L	77 g/L	63 g/L
Fragmentocytes	NA	neg.	neg.	pos.
Platelet count (150–350 × 10^9^/L)	374 × 10^9^/L	114 × 10^9^/L	43 × 10^9^/L	51 × 10^9^/L
*Chemistry*				
Potassium (3.5–5.1 mmol/L)	3.69 mmol/L	5.49 mmol/L	4.92 mmol/L	4.83 mmol/L
Phosphate (0.87–1.45 mmol/L)	1.42 mmol/L	2.24 mmol/L	1.97 mmol/L	2.41 mmol/L
Creatinine (62–106 μmol/L)	71 μmol/L	907 μmol/L	1,006 μmol/L	985 μmol/L
Urea (<8.3 mmol/L)	5.6 mmol/L	31.7 mmol/L	29.6 mmol/L	32.3 mmol/L
LDH (135–225 U/L)	NA	151 U/L	241 U/L	227 U/L
Haptoglobin (0.3–2.0 g/L)	NA	0.47 g/L	<0.1 g/L	NA
Total bilirubin (<21.0 μmol/L)	4 μmol/L	5.0 μmol/L	7.1 μmol/L	NA
Albumin	27 g/L	NA	43 g/L	42 g/L
Total cholesterin (<5 mmol/L)	9.52 mmol/L	NA	2.9 mmol/L	NA
Cholesterin-HDL (>1 mmol/L)	1.47 mmol/L	NA	1.3 mmol/L	NA
Cholesterin-LDL	7.57 mmol/L	NA	1.4 mmol/L	NA
Triglyceride (<2 mmol/L)	1.63 mmol/L	NA	1.6 mmol/L	NA
*Complement and ADAMTS-13*				
C3 (0.9–1.8 g/L)	1.05 g/L	0.81 g/L	0.74 g/L	NA
C4 (0.1–0.4 g/L)	0.19 g/L	0.20 g/l	0.21 g/L	NA
ADAMTS-13 activity	NA	NA	NA	100%
**Urine**				
Prot/Creat ratio (<11.3 mg/mmol)	586 mg/mmol	230 mg/mmol	NA	NA

* *NA, non-available*.

In June 2019, after his return to Switzerland, he complained about fatigue, epigastric pain, and a dizzy sensation. Blood pressure was 171/106 mmHg. No edema was noticed. In contrast to the findings 7 years earlier, the lipid profile was normal. The laboratory results showed progressive thrombocytopenia and coombs-negative hemolytic anemia with fragmentocytes (Table 1), and the diagnosis of thrombotic microangiopathy (TMA) was made. At this time, C3 levels were reduced. ADAMTS13 activity was 100% (Table 1). We started dialysis in July 2019, shortly after his first emergency consultation in our clinic. Due to a paracentral scotoma (left > right) fundoscopic exam was performed, which showed hypertensive retinopathy. A magnetic resonance imaging (MRI) of the brain was normal. A detailed, further analysis of the complement system revealed a functional complement defect (Table 2) and the presence of a homozygous aHUS risk allele for CFHR1, CFHR1^*^B (Table 3). Factor H antibodies were negative. Currently, the patient is listed for renal transplantation. We plan to treat the patient with the C5 inhibitor eculizumab during the first 3–6 months after transplantation. Close monitoring of the complement activity, i.e., CH50 test and C3 levels, will be required. Also, protocol biopsies will help us to detected aHUS and LPG recurrence at an early stage. Depending on posttransplant lipid levels and the results of protocol biopsies, we plan to treat the patient with fibrates as their use can induce clinical remission in some patients with LPG (3).

**Table 2:** C5b-9 complement deposition on human microvascular endothelial cells (HMEC-1).

**HMEC cells**	**C5b-9 deposits**	**Normal values**
Resting	159%	<150%
Activated	207%	

*The table shows the results of an ex vivo functional complement assay. In short, confluent human microvascular endothelial cells (HMEC-1) were maintained unstimulated or activated with 10 μmol/l ADP for 10 min and then incubated with the patient's serum or control serum for 2 of 4 h, respectively. HMEC-1 were fixed and stained with rabbit anti-human C5b-9 antibody followed by a fluorescein isothiocyanate–conjugated secondary antibody. Fluorescent staining on the cell surface was acquired through confocal microscopy. The stained area was evaluated using the Image J software. Results are expressed as a percent of staining in relation to the control serum (12)*.

**Table 3:** Complement system: sequencing analysis by Next-Generation Sequencing (NGS).

**Genes**	**Result**
**Complement factor H (CFH)**including the non-coding portion upstream of the gene containing the variant **rs3753394**	Normal
**Membrane cofactor protein (MCP) or CD46**including the non-portion downstream of the gene containing the variant **rs7144**	Normal
**C3**	Normal
**Complement factor I (CFI)**	Normal
**Complement factor B (CFB)**	Normal
**Thrombomodulin (THBD)**	Normal
**ADAMTS13**	Normal
**Complement factor H related 1-5 (CFHR1-5)**	Homozygous risk allele CFHR1^*^B
**C5**	Normal
**Diacylglycerol kinase** **ε** **(DGKE)**^**#**^	Normal
Methylmalonic aciduria and homocystinuria, cb1C type (MMACHC)^*^	Normal

The table shows the results of analyses by Next Generation Sequencing (NGS) of complement genes and genes related to secondary complement disorders (13, 14). The patient was homozygous for the aHUS risk allele CFRH1^*^B (9). For C5 the patient was found wildtype (normal) for the two polymorphisms c.2672G>A (rs56040400) and c.2671G>T (rs373359894), which are associated with a poor response to eculizumab (15).

# *Loss of DGKE function results in a prothrombotic state, which may lead to TMA/aHUS (16)*.

* *MMACHC is an inborn error of cobalamin metabolism. The consecutive accumulation of homocysteine may trigger endothelial injury and TMA/aHUS (17)*.

## Discussion

We present a case of a young, swiss-indonesian man with LPG. The initial manifestation was hypertension and nephrotic syndrome. Seven years after the diagnosis of LPG, he presented himself with end-stage renal disease. At this time, the diagnosis of TMA was made. A detailed investigation of the complement system found the homozygous aHUS risk allele CFHR1^*^B, and a functional complement assay demonstrated an overactivation of the complement system.

LPG is an autosomal dominant disease with variable penetrance caused by genetic alterations of the APOE gene (1). In the presence of pathognomonic glomerular capillary lipid thrombi, the diagnosis of LPG is straightforward. However, expansive forces by accumulating lipoproteins can result in mesangiolysis and glomerular basement membrane duplication mimicking membranoproliferative glomerulonephritis (MPGN) (6). LPG may also mimic TMA (18).

In our patient, we found a 9-bp deletion in exon 4 (c.480_488del), resulting in a 3-amino acid deletion, which was previously associated with LPG (10, 11). According to a review including the majority of patients with LPG (117 cases, all with sequencing data of the APOE gene), the specific mutation found in this case was previously discovered in China (5 cases) and Japan (2 cases) (1). As the Chinese are the most significant ethnic minority of foreign origin in Indonesia, it can be speculated that our patient's ancestors originate from China.

**Figure 2:**
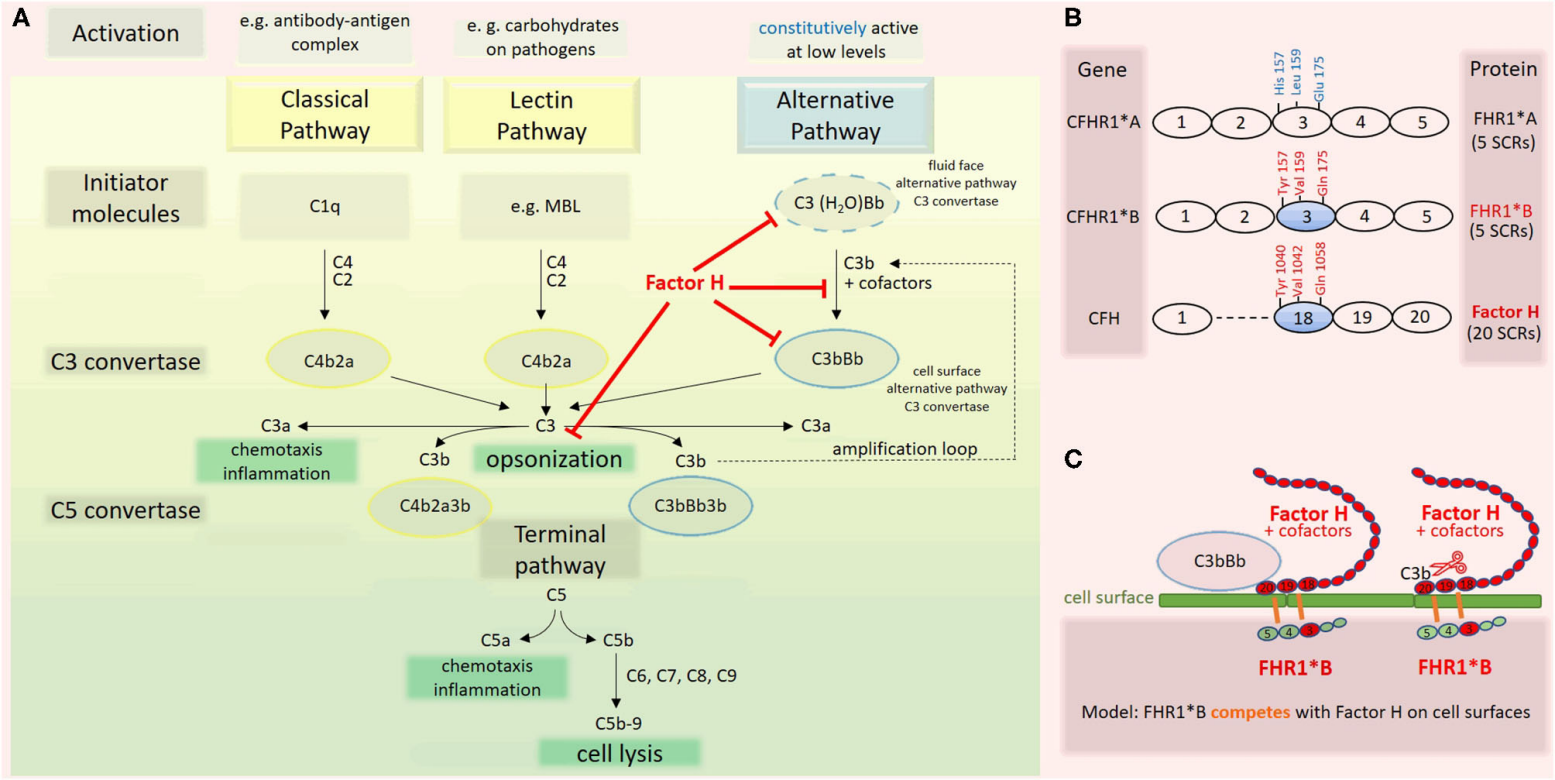
Complement activation cascade. Factor H and factor H related (FHR) 1 allotypes. **(A)** The complement system has three pathways (8). The classical pathway (CP) is triggered by antibody-antigen complexes interacting with C1q. The lectin pathway (LC) is triggered when lectins, e.g., mannose binding lectin (MBL), bind carbohydrates on surfaces. Both pathways lead to cleavage of C4 and C2, enabling the assembly of the C3 convertase C4b2a. The alternative pathway (AP) is constitutively active at low levels due to the spontaneous transformation of C3 to C3(H_2_O). This product is functionally C3b-like and allows the formation of the fluid phase C3 convertase C3(H_2_O)Bb. The latter cleaves C3 and enables the formation of the cell surface alternative pathway C3 convertase C3bBb. Factor H, the master regulator of the AP, interferes with this pathway at multiple levels (red). Assembly of the C5 convertases C4b2a3b and C3bBb3b initiates the last phase of the complement cascade, which is identical for all three pathways. **(B)** CFHR1 encoding FHR1 is a member of the factor H gene family. FHR1 comprises five short consensus repeats (SCRs) with different degrees of identity with homologous domains of factor H. The allotype CFHR1*B is associated with an increased risk for aHUS if present in homozygosity. FHR1*B harbors the amino acids Tyr at position 157, Val at 159, and Gln at 173 in SCR3, which are identical to those in SCR 18 of factor H (9). **(C)** Factor H is composed of 20 SCR domains arranged like beads on a string. On cell surfaces, factor H induces dissociation of surface-bound C3bBb and inactivation of C3b. The high sequence similarity of FHR1*B to factor H may lead to a competition between factor H and FHR1*B, a consecutive decrease of the functional activity of factor H and thus predisposing to aHUS (9).

This case with LPG has several interesting aspects. Dyslipidemia was no more present when the patient returned to Switzerland, and he was off lipid-lowering therapy. This suggests that the severe nephrotic syndrome at the initial presentation contributed to the altered lipid profile. Our patient may belong to a subgroup of individuals with LPG and dyslipidemia, which manifests only under specific circumstances. Furthermore, over the course of the disease, the patient developed TMA. In the literature, we found only one TMA report in a patient with LPG, attributed to malignant hypertension (19). Hypertension may have contributed to TMA in our patient as well. However, the reduced C3 levels suggest an overactivation of the alternative complement pathway as an underlying mechanism. This is further supported by the findings of an *ex vivo* assay with the patient's serum, which evoked increased binding of the terminal complement complex C5b-9 to resting and activated human endothelial cells (Table 3). Complementary, genetic analyses of the complement system found homozygosity for CFHR1^*^B (Figure 2B), a finding strongly associated with aHUS (9). A gene conversion event between the CFHR1 and CFH genes resulted in CFHR1^*^B, and functionally CFHR1^*^B is thought to compete with the complement regulatory factor H on cell surfaces (9) (Figure 2C). Interestingly, patients deficient in CFHR1 and CFHR3 have an increased risk for aHUS also. Their susceptibility is associated with factor H antibodies (9), which in turn may be due to modulation of B cell activation (20).

We do not know what triggered aHUS in this patient and to which extent aHUS accelerated renal function decline. However, we obtained a laboratory result from Indonesia showing thrombocytopenia 2 months before the patient returned to Switzerland. This indicates that aHUS may have contributed to the renal damage over a prolonged period.

Potentially, the APOE gene mutations associated with LPG may facilitate aHUS in a patient at risk, as apoE structural deformities caused by APOE mutations facilitate aggregation of apoE. These aggregates may not only directly contribute to the onset of LPG (4) but to the onset of aHUS also. As our patient's renal biopsy did not show any signs of TMA, the postulated apoE aggregates alone seem to be insufficient to trigger aHUS in this case. The lack of TMA signs in the renal biopsy (Figures 1A,B) makes it also unlikely that TMA triggered the onset of LPG. Additional factors such as hypertension and, further, unknown risk factors must have contributed to this patient's disease course.

## Conclusion

To the best of our knowledge, this is the first report of aHUS in a patient with LPG with a documented dysfunction of the complement system and an associated genetic risk factor.

## Data Availability Statement

The datasets presented in this study can be found in online repositories. The names of the repository/repositories and accession number(s) can be found in the article/Supplementary Material.

## Ethics Statement

Written informed consent from the patient has been obtained for the publication of any identifiable data.

## Author Contributions

AJ initiated the case study, the APOE sequencing, the detailed analysis of the complement system, and draw Figure 2. LK collected all data. LK and AJ wrote the manuscript. JS performed sequencing of APOE, analyzed the data, and reviewed the manuscript. HH performed histological analyses leading to the diagnosis of LPG, provided images, and reviewed the manuscript. PH-M helped with the preanalytical handling of blood samples for the complement analysis and reviewed the manuscript. EB was involved in the complement analysis and reviewed the manuscript. TN was responsible for the patient's initial workup leading to the diagnosis of LPG and reviewed the manuscript. All authors read and approved the submitted version of the manuscript.

## Conflict of Interest

The authors declare that the research was conducted in the absence of any commercial or financial relationships that could be construed as a potential conflict of interest.
